# Minimally invasive surgical repair of accessory mitral valve tissue: A case report

**DOI:** 10.1016/j.ijscr.2020.05.080

**Published:** 2020-06-09

**Authors:** Dinh Nguyen, Anh Vo, Chuong Pham, Trang Nguyen, Thanh Vu, Khoi Le, Bac Nguyen

**Affiliations:** a Department of Cardiovascular Surgery, University Medical Center, University of Medicine and Pharmacy at Ho Chi Minh City, Ho Chi Minh City, Viet Nam; b Department of Surgery, University of Medicine and Pharmacy at Ho Chi Minh City, Ho Chi Minh City, Viet Nam

**Keywords:** Accessory mitral valve tissue, Left ventricular outflow tract obstruction, Minimally invasive approach

## Abstract

**Introduction:**

Accessory mitral valve tissue is a rare congenital disease of the mitral valve. It is usually associated with other cardiac malformations and/or left ventricular outflow tract obstruction. More than 2/3 of patients were diagnosed in their childhood. Treatment can be conservative or surgical. The suitable timing for surgery remains controversial, some authors suggest early intervention to prevent devastating complications.

**Case Report:**

We report a case of an adult patient with accessory mitral valve tissue causing left ventricular outflow tract obstruction, who was treated surgically via the right minithoracotomy.

## Highlights

• Rare condition requiring early intervention to prevent thromboembolic events.
• Minimally invasive approach provides excellent exposure.
• Differentiation with mitral valve aneurysm is essential.

## 1 Introduction

Accessory mitral valve tissue (AMVT) is a rare congenital condition and sometimes associates with left ventricular outflow tract obstruction (LVOTO). The mechanism of this obstruction is direct bulging into LVOT of redundant anterior leaflet tissue, which increases the pressure gradient through the narrow path [5]. Roughly around 70 % cases are diagnosed during childhood, due to the symptoms related to LVOTO [10]. The condition can be treated conservatively, surgery should be indicated in patients with severe LVOTO, severe mitral valve regurgitation or in patients undergoing surgery for other cardiac malformations. The outcome could be even more beneficial if a minimally invasive approach is applied. We report a case of an adult patient with AMVT successfully treated via right minithoracotomy.

The work has been reported in line with the SCARE criteria [1].

## 2 Presentation of case

**Fig. 1:**
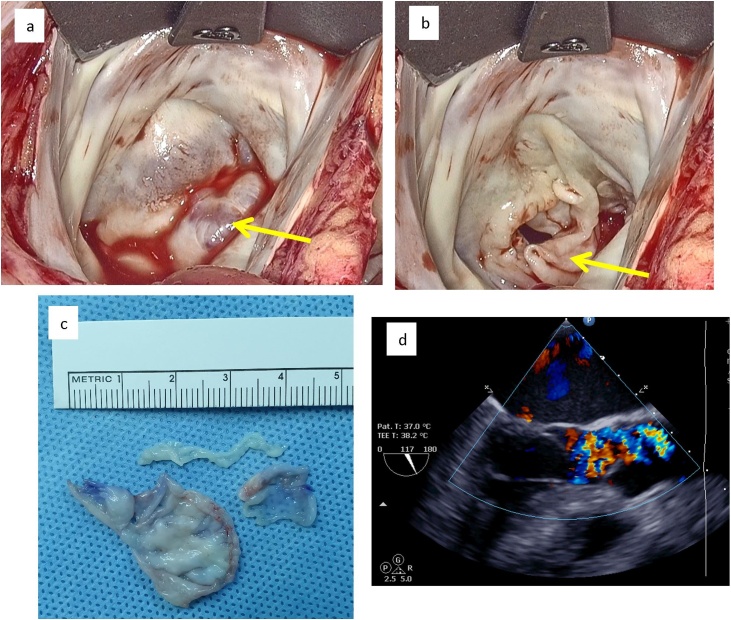
The anterior leaflet aneurysm outpouching to the LVOT (left), aliasing jet through the LVOT and the regurgitant jet of the mitral valve on 3-chamber view (right).

A 39-year-old male with the history of dyspnea on exertion was admitted to our department. The patient had a worsening dyspnea in a 4-month period. On admission, he presented with mild orthopnea without history of paroxysmal nocturnal dyspnea. Clinical examination revealed a regular heart rate of 80 bpm, blood pressure of 110/60 mmHg and a 4/6 holosystolic murmur at the apex, radiating to the left axilla. Another 3/6 systolic murmur was also discovered at the left 3rd intercostal space. 2D transthoracic echocardiography (TTE) showed an 18 × 15 mm anterior leaflet aneurysm. This structure protruded into the left ventricular outflow tract (LVOT), caused severe LVOTO. The pressure gradient through the LVOT was 97/49 mmHg (Continuous wave Doppler measurement). Severe mitral regurgitation was also detected, the vena contracta of the mitral regurgitant jet was 8.1 mm (Fig. 1). Left ventricular ejection fraction was 64 % (Simpson-Biplane). No aortic valve lesion and other cardiac malformation were found on preoperative TTE.

**Fig. 2:**
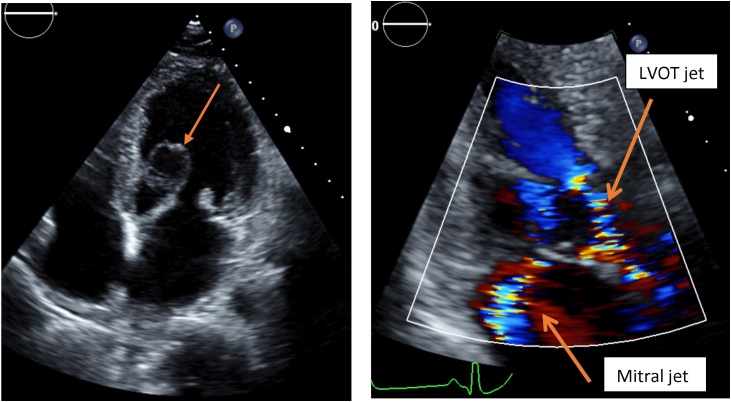
The AMVT with water test (a) and without water test (b), excised AMVT (c), postoperative TEE showed competent mitral valve (d).

The patient underwent minimally invasive mitral valve repair. Cardiopulmonary bypass was established via the right femoral vessels. A 5 cm right thoracotomy through the 4th- intercostal space was performed. The left atrial was entered and the mitral valve was exposed. The AMVT was found on the A2 part of the anterior leaflet, near the valvular free edge. The pouch was then exposed and excised (Fig. 2). The remaining leaflet was reattached using interrupted 5.0 sutures. Ring annuloplasty was performed to reinforce the mitral annulus.

Left atrium was closed and the CPB was weaned as usual. TEE showed a competent mitral valve and the LVOT was clear with a mean gradient of 6 mmHg. After surgery, the biopsy showed no-specific pathological findings.

The postoperative phase was uneventful. The patient was extubated 6 h later with the support of low-dose dobutamine. He was discharged at postoperative day 5. At 3-month follow-up, he has fully recovered. TTE showed a trivial mitral regurgitation with no LVOT obstruction.

## 3 Discussion

AMVT is generally diagnosed in the childhood due to its association with other cardiac malformations and/or a symptomatic LVOTO. As a result, adult cases of AMVT is very rare [8]. In this case, the patient developed the symptom in his adulthood owing to the mitral regurgitation and the LVOTO. The appropriate timing for surgical resection of the AMVT remains controversial. According to Prift E. et al., the excision of the AMVT is recommended when the patient is symptomatic [7]. However, a case report showed a cerebral thromboembolic event in a young woman caused by thrombosis in the pouch. Hence, and the authors advocated surgery at the time of diagnosis to prevent future devastating complications [9].

The benefits of minimally invasive mitral valve surgery have been proved in the literature [2]. This is becoming the new trend for mitral valve surgery in many centers. Through the right minithoracotomy, the AMVT could be clearly exposed and the procedure seems to be simple. With the combination of the minimally approach and the simplicity of the AMVT resection, we support early intervention to avoid neurologic events in the youngsters.

AMVT need to be differentiated with mitral valve aneurysm (MVA), a rarely reported disease. It is a complication of aortic valve infective endocarditis [6]. TTE usually shows an outpouching of the anterior leaflet into the left atrium with systolic expansion and diastolic collapse. Rupture or perforation of the aneurysm is one of the severe complications because of the subsequent acute mitral valve regurgitation [4]. Small-size pouches can be treated conservatively and surgery is reserved for large unruptured aneurysms or in the setting of rupture or perforation [3]. TTE and TEE are essential for diagnosis and decision making. Aortic valve should be carefully studied to exclude any endocarditis proof of the valve. The direction of the pouch (toward the left atrium or the left ventricle) also helps to make the discrimination.

## 4 Conclusion

In conclusion, AMVT is a rare disease which needs to be treated early to prevent future neurological complications. Minimally approach via the right minithoracotomy provides an excellent exposure and facilitates the procedures. This condition should be distinguished with the MVA due to different pathophysiology and treatment.

## Declaration of competing Interest

All authors declare no conflict of Interests.

## Sources of funding

We do not receive funding from any source.

## Ethical approval

The case was part of the data on minimally invasive mitral valve surgery, approved by the ethical board of the University of Medicine and Pharmacy at Ho Chi Minh City, number 141/DHYD-HDDD, on April 11th 2018.

## Consent

Written informed consent was obtained from the patient for publication of this case report and accompanying images. A copy of the written consent is available for review by the Editor-in-Chief of this journal on request.

## Author contribution

Dinh Nguyen, Anh Vo, Chuong Pham, Thanh Vu: Surgeons.

Khoi Le, Trang Nguyen: Echocardiologist.

Bac Nguyen: Administration.

## Registration of research studies

1 Name of the registry:
2 Unique identifying number or registration ID:
3 Hyperlink to your specific registration (must be publicly accessible and will be checked

## Guarantor

Dinh Nguyen.

## Provenance and peer review

Not commissioned, externally peer-reviewed.

## References

[1] Agha R.A., Borrelli M.R., Farwana R. (2018). The SCARE 2018 statement: updating consensus surgical CAse REport (SCARE) guidelines. Int. J. Surg..

[2] Cheng D.C., Martin J., Lal A. (2011). Minimally invasive versus conventional open mitral valve surgery: a meta-analysis and systematic review. Innovations (Phila).

[3] Gin K.G., Boone J.A., Thompson C.R. (1993). Conservative management of mitral valve aneurysm. J. Am. Soc. Echocardiogr..

[4] Janardhanan R., Kamal M.U., Riaz I.B. (2016). Anterior mitral valve aneurysm: a rare sequelae of aortic valve endocarditis. Echo Res. Pract..

[5] Manganaro R., Zito C., Khandheria B.K. (2014). Accessory mitral valve tissue: an updated review of the literature. Eur. Heart J. Cardiovasc. Imaging.

[6] Moretti M., Buscaglia A., Senes J. (2018). Anterior mitral valve aneurysm is an uncommon complication of aortic valve infective endocarditis: a case report. Am. J. Case Rep..

[7] Prifti E., Bonacchi M., Frati G. (2002). Accessory mitral valve leaflet in an adult with coronary artery disease. J. Cardiovasc. Surg. (Torino).

[8] Tennichi T., Taniguchi T. (2019). Accessory mitral valve tissue that caused a left ventricular outflow tract obstruction: a case report. JA Clin. Rep..

[9] Yetkin E., Turhan H., Atak R. (2003). Accessory mitral valve tissue manifesting cerebrovascular thromboembolic event in a 34-year-old woman. Int. J. Cardiol..

[10] Yuan S.M., Shinfeld A., Mishaly D. (2008). Accessory mitral valve tissue: a case report and an updated review of literature. J. Card. Surg..

